# Resistance genomics and molecular epidemiology of high-risk clones of ESBL-producing *Pseudomonas aeruginosa* in young children

**DOI:** 10.3389/fcimb.2023.1168096

**Published:** 2023-05-24

**Authors:** Sandip Patil, Xiaowen Chen, Shaowei Dong, Huirong Mai, Bruno Silvester Lopes, Sixi Liu, Feiqiu Wen

**Affiliations:** ^1^ Department of Haematology and Oncology, Shenzhen Children’s Hospital, Shenzhen, China; ^2^ Paediatric Research Institute, Shenzhen Children’s Hospital, Shenzhen, China; ^3^ School of Health and Life Sciences, Teesside University, Middlesbrough, United Kingdom; ^4^ National Horizons Centre, Teesside University, Darlington, United Kingdom

**Keywords:** *P. aeruginosa*, ESBLs, antimicrobial susceptibility, mlst, PBRT, MOB typing

## Abstract

**Introduction:**

The emergence of multidrug-resistant *Pseudomonas aeruginosa* poses a global threat, but the distribution and resistance profiling are unclear, especially in young children. Infections due to *P. aeruginosa* are common, associated with high mortality, and increasingly β-lactam drug resistant.

**Methods:**

We studied the molecular epidemiology and antibiotic resistance mechanisms in 294 clinicalisolates of *P. aeruginosa* from a pediatric hospital in China. Non-duplicate isolates were recovered from clinical cases and were identified using an API-20 kit followed by antimicrobial susceptibility testing using the VITEK®2 compact system (BioMerieux, France) and also by broth dilution method. In addition, a double-disc synergy test for the ESBL/E-test for MBL was performed. The presence of beta-lactamases, plasmid types, and sequence types was determined by PCR and sequencing.

**Results:**

Fifty-six percent (*n* = 164) of the isolates were resistant to piperacillin–tazobactam, followed by cefepime (40%; *n* = 117), ceftazidime (39%; *n* = 115), imipenem (36%; *n* = 106), meropenem (33%; *n* = 97), and ciprofloxacin (32%; *n* = 94). Forty-two percent (n = 126) of the isolates were positive for ESBL according to the double-disc synergy test. The blaCTX-M-15 cephalosporinase was observed in 32% (n = 40/126), while 26% (n = 33/126) werepositive for blaNDM-1 carbapenemase. Aminoglycoside resistance gene *aac(3)IIIa*was observed in 16% (n = 20/126), and glycylcyclines resistance gene tet(A) was observed in 12% (n = 15/126) of the isolates. A total of 23 sequence types were detected, including ST1963 (12%; n = 16), followed by ST381 (11%; *n* = 14), ST234 (10%; *n* = 13), ST145 (58%; *n* = 10), ST304 (57%; *n* = 9), ST663 (5%; n = 7), and a novel strain. In ESBL-producing *P. aeruginosa*, 12 different Incompatibility groups (Inc) were observed, the most common being IncFI, IncFIS, and IncA/C. The MOBP was the most common plasmid type, followed by MOBH, MOBF, and MOBQ.

**Discussion:**

Our data suggest that the spread of antibiotic resistance is likely due toclonal spread and dissemination of different clinical strains of *P. aeruginosa* harbouring different plasmids. This is a growing threat in hospitals particularly in young children which needs robust prevention strategies.

## Introduction

*Pseudomonas aeruginosa* (*P. aeruginosa*) is a major medically important opportunistic pathogen that causes a variety of clinical conditions (Qin et al., 2022). It is best known for causing lung infection among cystic fibrosis patients and critical infections among immunocompromised patients, patients with severe burns, catheterized patients, and other indoor patients, such as those with conditions (Mateu-Borrás et al., 2022; Verhoeve et al., 2022). *P. aeruginosa* also causes blood infections, respiratory tract infections (RTIs), skin infections, urinary tract infections (UTI), and surgical site infections/wound infections (Kothari et al., 2022). A restricted choice of antimicrobial agents, such as cephalosporins and carbapenem, has reliable activity against *P. aeruginosa* infections (Ibrahim et al., 2020). The effective treatment of such opportunistic infections remains a significant challenge for clinical practitioners globally, including in developing nations such as China (Raman et al., 2018). Carbapenem-resistant *P. aeruginosa* was listed as a critical priority pathogen by the World Health Organization (WHO) in 2017 owing to its ability to easily acquire antibiotic resistance determinants and increasing resistance to the most commonly used antibiotics such as carbapenem, cefepime, and ceftriaxone (WHO, 2017). The ability of *P. aeruginosa* to withstand the effect of commonly used antibiotics has been escalating exponentially over the years (Kunz Coyne et al., 2022). Resistance development can occur *via* specific gene mutations or by the acquisition of antibiotic-resistant genes. Competence mechanisms such as horizontal gene transfer through mobile elements aid in the process of multidrug resistance (MDR) in bacteria, which can further evolve into extreme drug resistance (XDR) (Tenover et al., 2022). The prevalence of MDR *P. aeruginosa* is rising globally, with a range of 15%–30% worldwide, including in China (Dou et al., 2017; Horcajada et al., 2019; Al Hamdan et al., 2022; Mohamed et al., 2022). The MDR *P. aeruginosa* may possess enzymes that can hydrolyze the majority of the β-lactam drugs, examples of which include enzymes such as class A (KPC and GES), class B (IMP, NDM, and SPM), and class D (OXA-48 β-lactamases) (Glen and Lamont, 2021). In 2018, difficult-to-treat resistance (DTR) *P. aeruginosa* was observed in the United States and was resistant to a variety of drugs, including piperacillin–tazobactam, ceftazidime, cefepime, aztreonam, meropenem, imipenem, ciprofloxacin, and levofloxacin, and it was linked to high mortality (Tamma et al., 2022). *P. aeruginosa* develops resistance successfully through genetic mutations or overexpression of specific efflux pump genes like *mexAB-OprM, mexXY–OprM*, and *mexCD–OprJ*. These genes can typically reduce susceptibility not only to β-lactam antibiotics but also to quinolones, macrolides, novobiocin, chloramphenicol, tetracyclines, and lincomycin (Pesingi et al., 2019). Plasmids can carry antibiotic resistance genes such as *bla*_CTX-M,_ *bla*_NDM_, *bla*_KPC_, and *bla*_SHV_ and facilitate their efficient transfer within and between bacterial species. This plays a crucial role in disseminating antibiotic resistance among strains of *P. aeruginosa* (Patil et al., 2019a). Based on the critical property of MDR *P. aeruginosa*, molecular epidemiological investigations have become essential in the form of surveillance studies to tackle any local or global outbreaks and monitor the long-term progress of predominant MDR clones (Pulusu et al., 2022). PCR-based multi-locus sequence typing (MLST) has been used as an epidemiological tool, as it has been proven to be efficient in studying the population structure of *P. aeruginosa* (Magalhães et al., 2020). Several highly infectious strains of *P. aeruginosa*, including ST175, ST235, and ST463, have recently emerged as hypervirulent clones of *P. aeruginosa*. This is most likely due to a unique combination of multiple drug resistance and virulence (Hosu et al., 2021). Concerning the possible risk factors of MDR *P. aeruginosa*, indiscriminate use of antimicrobials in the healthcare settings and agricultural sector promotes the development of antibiotic resistance. Thus, it is essential to prioritize rapid and frequent surveillance, reduce unnecessary prescribing in patients, and limit the usage of antimicrobials in agriculture to break the chain of disease transmission. The present study aims to identify ESBL-encoding genes, antibiotic susceptibility, clonal spread, and plasmid-associated genes involved in the dissemination of antibiotic resistance in *P. aeruginosa* isolated from pediatric patients in Shenzhen, China.

## Materials and methods

This study was conducted in accordance with the guidelines of the Declaration of Helsinki and approved by the Institutional Ethics Committee of Shenzhen Children’s Hospital, reference number: 2018 (013), 3rd Sept 2018, which complies with international ethical standards. All experiments were compliant with the hospital’s biosafety regulations. *P. aeruginosa* isolates were collected from various clinical conditions, such as fever, cough, wound swelling, UTI, RTI, bacteremia, and ear pain, from different clinical samples such as blood, urine, pus, stool, and pleural secretion. All isolates were obtained between 1 February 2021 and 28 February 2022 from the central microbiology laboratory of Shenzhen Children’s Hospital as part of a routine hospital investigation. The Ethics Committee of Shenzhen Children’s Hospital determined that patients’ or guardians’ consent was not required due to the retrospective nature of the study. Personal information such as names were not used for research purposes, and data were kept confidential and compliant with the Declaration of Helsinki; therefore, written consent was not required

### Clinical sampling and identification of isolates

A total of 294 non-repetitive isolates were collected (one isolate from one patient), and other demographic characteristics of the patients were collected from the electronic data record room, including sex, age, hospitalized department, and treatment of antibiotics. All specimens were cultured on a blood agar and MacConkey’s agar, but blood and sputum were cultured on the chocolate agar. Further suspected colonies transferred to cetrimide agar and observed for colony morphology, motility, and Gram stain. Furthermore, they identified using the VITEK^®^2 compact system (BioMerieux, France) and confirmed by API-20 and 16S-RNA sequencing (Sangon Biotech, Shanghai). The genomic DNA was extracted using the QIAamp DNA Mini Kit (Qiagen) according to the manufacturer’s instructions. The quality of the extracted DNA was assessed by measuring the absorbance at 260- and 280-nm wavelengths. The cultures were kept for 3 days (SD 2.83), followed by storage in 30% glycerol stock at −80°C for further investigation. The full length of the 16S rRNA gene was amplified by conventional PCR by using universal primers as per the protocol described earlier (Lin et al., 2022). The PCR reaction total volume was 20 µl, containing 1 µl (30 ng) of genomic DNA, 10 µl of 2X Master Mix, 0.4 µl (20 pmol) of each forward and reverse primer, and 8.2 µl of nuclease-free water. Thermocycler conditions were set at 94^°^C for 5 min, followed by 35 cycles of denaturation at 94^°^C for 30 s, annealing at 55^°^C for 30 s, and extension at 72^°^C for 1 min, followed by a final extension at 72^°^C for 5 min. Deionized sterile distilled water was used as the control, while *P. aeruginosa* ATCC 27853 DNA was used as a positive control. All amplified products were run on 1.8% agarose gel and stained with ethidium bromide. The products were purified using the QIAquik gel extraction kit as per the manufacturer’s instructions. The purified products were sequenced (forward and backwards) by Sangon Biotech, Shanghai. The sequenced results were aligned with published reference sequences in the NCBI BLAST database and confirmed as *P*. *aeruginosa*. All statistical analyses were performed using chi-square two-tailed test, and *p*-values <0.05 were considered significant.

### Antimicrobial susceptibility and phenotypic detection of ESBL/MBL

The antimicrobial susceptibility test (AST) was performed on all 294 confirmed isolates using the VITEK^®^2 compact system (BioMerieux, France) with the standard AST09 card (software version 9.01). In addition, minimum inhibitory concentration (MIC) was determined by the broth dilution method for the antimicrobial agent tested including aminoglycosides (amikacin and tobramycin); carbapenems (imipenem and meropenem); cephalosporins (ceftazidime and cefepime), fluoroquinolones (ciprofloxacin and levofloxacin), penicillins plus β-lactamase inhibitors (piperacillin–tazobactam and ticarcillin/clavulanate), glycylcycline (tigecycline), cefoperazone/sulbactam, and chloramphenicol. Isolates showing intermediate antimicrobial-resistant strains were considered resistant strains for statistical analysis (Patil et al., 2022). The AST results were accurately interpreted according to the Clinical and Laboratory Standards Institute (CLSI) breakpoints 2017 and European Committee on Antimicrobial Susceptibility Testing (EUCAST) (CLSI, 2019; EUCAST, 2020). The classification of the MDR phenotype was performed as described previously by Magiorakos et al., in which MDR was defined as resistance to at least one agent in three or more antimicrobial groups, and XDR was defined as those that are resistant to at least one agent in all but two antimicrobial categories (Magiorakos et al., 2012). The ESBL production was initially determined by using the VITEK^®^2 compact system (BioMerieux, France). However, for better accuracy and precision, the results were confirmed using a double-disc synergy test (DDST). The DDST includes a disc of amoxicillin–clavulanic acid (20/10 mcg), ceftriaxone (30 mcg), ceftazidime (30 mcg), and cefotaxime (30 mcg). In addition, metallo-beta-lactamase (MBL) production was determined by using MBL-Etest (IPM-EDTA; AB Biodisk) for ESBL-producing *P. aeruginosa*. All ESBL production detection discs and E-strips were placed properly on a seeded Mueller–Hinton agar plate with standard inoculum (Reference to 0.5 McFarland) using a cotton swab. The incubation was at 37°C/24 hours. The enhancement of the zone of inhibition when testing with amoxicillin–clavulanic acid with one of the four ESBL production detection discs suggests the presence of ESBL. A *Salmonella enterica* ST34 strain SP-15-127 was used as a positive control, which had been previously characterized in our laboratory as a strain positive for *bla*_CTX-M-15_ ESBL gene (Patil et al., 2022) hand *P. aeruginosa* ATCC 27853, and *E*. *coli* ATCC25922 were used as negative controls. The ESBL production results were analyzed according to the EUCAST guidelines.

### Detection of β-lactamase-encoding genes

Isolates classified as β-lactamase producers (*n* = 126) were tested by the PCR assay to determine the β-lactamase-encoding genes such as *bla*_SHV_, *bla*_GES_, *bla*_TEM_, *bla*_CMY_, *bla*_VIM_, *bla*_KPC,_ *bla*_NDM_, *bla*_CTX-M_, *bla*_OXA-48_, and *bla*_IMP_; aminoglycoside resistance gene *aac(3)IIIa* and glycylcycline resistance *tetA*(A) were screened with primers as previously described and listed in **Supplementary Table 1** (Patil et al., 2019b). DNA sequencing was performed at Sangon Biotech Pvt Ltd, Shanghai, on the amplification of PCR-positive products, and the obtained results were compared and precisely aligned with published reference sequences using the online NCBI BLAST database to identify any specific resistant genes.

### Molecular typing

To uniquely determine the clonal relationship between the *P. aeruginosa* strains, we performed strain-typing based on seven housekeeping genes, *acsA, aroE, guaA, mutL, nuoD, ppsA*, and *trpE*, according to the instructions on the MLST strain typing website for *P. aeruginosa* (http://pubmlst.org/paeruginosa/). The primers for the amplification of the above housekeeping genes were adopted as described earlier by Curran et al. (2004) (**Supplementary Table 2**). PCR conditions were developed in our laboratory with a 20-µl amplification reaction mixture composed of 1 µl (30 ng) of genomic DNA, 10 µl of 2X Master Mix (Sangon Biotech), 0.4 µl (20 pmol) of each forward and reverse primer, and 8.2 µl of nuclease-free water. Thermocycler conditions were set at 94^°^C for 5 min, followed by 35 cycles of denaturation at 94^°^C for 30 s, annealing at 55^°^C for 30 s, and extension at 72^°^C for 1 min, followed by a final extension at 72^°^C for 5 min. Deionized sterile distilled water was used as the control, while *P. aeruginosa* ATCC 27853 DNA was used as a positive control. All PCR products were run through 1.5% agarose gel with ethidium bromide, and specific products were purified using the gel extraction kit. The purified products were sequenced by Sangon Biotech, Shanghai. The sequences were analyzed using the MLST database (https://pubmlst.org/paeruginosa), and the sequence type (ST) was determined. The combinations of alleles that did not match were designated as a “new” ST. The correlation between ST types and antimicrobial drug resistance groups was established using the Gephi network analysis software. Additionally, we thoroughly analyzed the possible distribution of resistance-encoding genes among the 126 isolates of ESBL-producing *P. aeruginosa* based on their ST types. The data were visualized using PRISMA 8.0 software.

### Plasmid typing and conjugation assay

Plasmid DNA was isolated using the Invitrogen PureLink-HiPure plasmid filter miniprep standard kit as per the manufacturer’s instructions. The Plasmid incompatibility (Inc) group was determined by typically using PCR-based replicon typing (PBRT). Eighteen specific primer pairs were designed, based on the multiple comparative analysis of nucleotide sequence on the EMBL Gene Databank, for HI1, HI2, I1-Iγ, X, L/M, N, FIA, FIB, W, Y, P, FIC, A/C, T, FIIA_S_, F, K, and B/O replicons, and the results were analyzed as described earlier Carattoli et al, 2005. Amplicons were visualized by gel electrophoresis on a 1.8% agarose gel stained with ethidium bromide. The genomic DNA of the *E*. *coli* DH5α strain was used as the negative control. The degenerate primer MOB typing (DPMT) was detected by using the amplification of the 19 key pairs of primers that were encoded in 33 reference MOB relaxase genes. The primers and PCR assay protocol were as described earlier [Villa and Carattoli A (2020)]. All primers are listed in **Supplementary Table 2**. We conducted a resistance gene transfer experiment using the broth mating method for all isolates to access the location of the genetic environment of the ESBL-encoding genes and plasmid transfer frequency. We used streptomycin-resistant *E. coli* C_600_ as the recipient strain. Transconjugants were selected on Muller–Hinton agar. Antimicrobial susceptibility testing, a confirmatory test for the ESBL phenotype, and PCR detection were performed for the transconjugants using the same procedures as described above.

### Statistical analysis

The data were organized and inputted into an Excel database and analyzed using SPSS version 23.0. The statistical significance level was set at <0.05 for all statistical evaluation

## Results

This single-center child population study was conducted between 1 February 2021 and 28 February 2022, and it was primarily designed to obtain insights into the age-specific resistance phenotype of infectious diseases. In this research, 294 P*. aeruginosa* were recovered from different specimens such as urine (25%; *n* = 75), followed by sputum (17%; *n* = 49), stool (16%; *n* = 48), blood (14%; *n* = 43), abdominal secretion (8%; *n* = 23), throat swab (6%; *n* = 20), cerebrospinal fluid (4%; *n* = 12), pus (3%; *n* = 10), appendix (3%; *n* = 6), body secretion (2%; *n* = 5), and pleural fluid (1%; *n* = 3). Of the 294 isolates, 64% (*n* = 190) were from male subjects, while 46% (*n* = 104) were from female subjects; the chi-square statistical value was 0.019 (*p*-value = 0.88), indicating that there was no significant difference in infection based on a specific sex. The interquartile range (IQR) between ages 0 and 14 (*p-*value = 0.51) revealed that age was also not significant to *P. aeruginosa* infection (**Table 1**). We have confirmed the presence of *P. aeruginosa* strains based on their morphological characteristics, such as yellow-green or yellow-brown fluorescent pyoverdine, and the presence of large, smooth colonies on cetrimide agar. Microscopic examination also revealed the strains to be Gram-negative and motile rods. The morphological results were supported by the results of API20 and 16sRNA sequencing. The extracted DNA from *P. aeruginosa* had an OD_260_/OD_280_ ratio between 1.7 and 2.0; it was considered to be of good quality and was used in further molecular analyses.

**Table 1:** Participant characteristics and isolates details.

	ESBLs positive (n = 126)	ESBLs negative (n = 168)	p-value^*^
Age			
Mean (SD)	4.8 (4.2)	2.1 (2.1)	0.51
Median (IQR)	6.5 (1–6)	0.7 (1–6)	
Sex			
Male	82 (65%)	108 (64%)	0.88
Female	44 (35%)	60 (36%)	
Specimens			
Abdominal secretion	11 (8%)	12 (7%)	0.98
Appendix	2 (1%)	4 (2%)	
Blood	19 (15%)	24 (14%)	
CSF and others	5 (4%)	7 (4%)	
Pleural fluid	1 (4%)	2 (1%)	
Pus	8 (6%)	2 (1%)	
Body secretion	2 (1%)	3 (2%)	
Sputum	20 (15%)	29 (17%)	
Stool	20 (15%)	28 (16%)	
Throat swab	8 (6%)	12 (7%)	
Urine	30 (25%)	45 (27%)	
Departments			
Hematology	10 (8%)	26 (15%)	0.95
Nephrology	2 (2%)	5 (3%)	
Neonatology	4 (3%)	6 (4%)	
Respiratory	7 (6%)	11 (7%)	
PICU	16 (13%)	17 (10%)	
Surgery cardiac	40 (32%)	36 (21%)	
Urology	5 (4%0	7 (4%)	
Oncology	16 (13%)	28 (17%)	
OPD	10 (8%)	17 (10%)	
Emergency	16 (13%)	15 (9%)	

Data are number (n); percentage (%); standard deviation:(SD); interquartile range (IQR). We used the abdominal secretion, appendix, blood, CSF and other (cerebrospinal fluid and site unknown), pleural fluid, pus, body secretions, sputum, stool, throat, and urine specimens. ESBL = extended-spectrum β-lactamase; *p-values were obtained using an independent t-test for continuous data variables with two-tailed hypothesis. PICU, pediatric intensive care; OPD, outpatient department.

### Antibiotic sensitivity patterns and gene analyses

**Figure 1:**
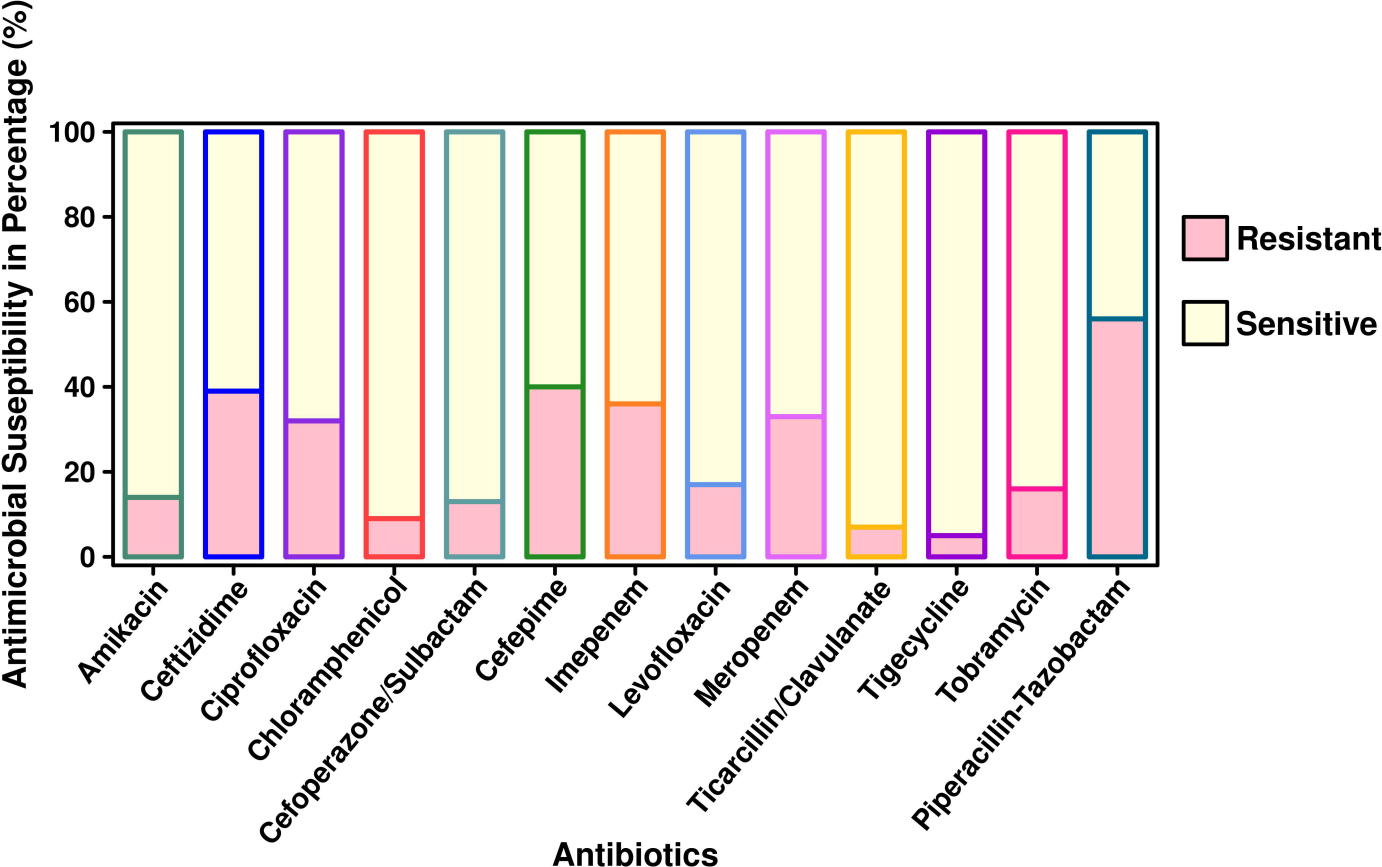
Antimicrobial susceptibility pattern of *P. aeruginosa* isolated from pediatric patients (*n* = 294).

**Figure 2:**
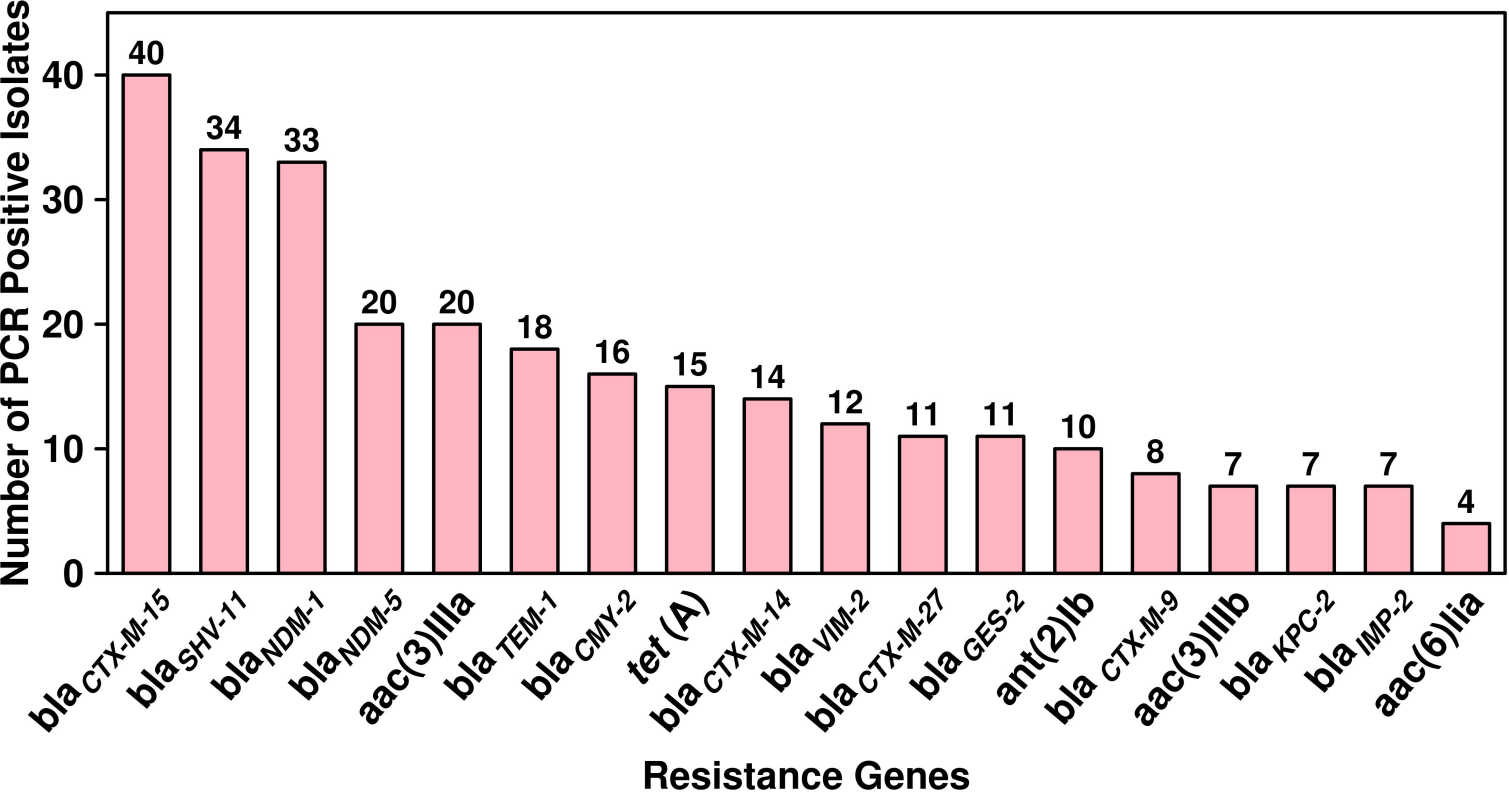
Dissemination and prevalence of antimicrobial resistance genes among ESBL-producing *P. aeruginosa* (*n =* 126).

The antibiotic susceptibility testing results of all the 294 isolates revealed that the highest resistance was observed for piperacillin–tazobactam (56%; *n* = 164), followed by cefepime (40%; *n* = 117), ceftazidime (39%; *n* = 115), imipenem (36%; *n* = 106), meropenem (33%; *n* = 97), ciprofloxacin (32%; *n* = 94), tobramycin (16%; *n* = 47), amikacin (14%; *n* = 41), cefoperazone/sulbactam (13%; *n* = 38), ticarcillin/clavulanate (7%; *n* = 20), and tigecycline (5%; *n* = 15). The specific details of the susceptibility profile and respective percentages are shown in **Figure 1**. The considerable majority of the *P. aeruginosa* isolates were resistant to cephalosporins, β-lactam inhibitor combination, quinolones, and carbapenems but relatively less resistant to aminoglycoside, chloramphenicol, and glycylcyclines (*p* < 0.001). The double-disc synergy test method for ESBL detection showed that 42% (*n* = 126) of isolates were able to hydrolyze cephalosporins, and on the contrary, 40% (*n* = 119) of isolates were ESBL positive with the VITEK^®^2 compact system. The chi-square statistical value was 0.34 (*p*-value is 0.55), precisely indicating that there was no significant difference in both methods. Therefore, *n* = 126 (42%) β-lactamase-producing isolates were selected for further investigation. Among ESBL-positive isolates, only 10% (*n* = 12) showed MBL production. We observed that isolates from the surgical department had a high number of ESBL-producing *P. aeruginosa* at approximately 32% (*n* = 40/126), followed by the cancer department at 20% (*n* = 26/126), emergency and ICU at 13% (*n* = 16/126), and outpatient department at 8% (*n* = 10/126), and a lower number of cases from the respiratory department (6%; *n* = 7/126) and the urology department (4%; *n* = 5/126). According to the specimen, high numbers of ESBL-producing *P. aeruginosa* were recovered from urine at 25% (*n* = 30/126), 15% (*n* = 20/126) from sputum and stool, and 14% (*n* = 19/126) from blood. The ESBL-producing *P. aeruginosa* resistance phenotype data revealed that 50% (*n* = 58/126) showed MDR phenotype while 7% (*n* = 9/126) showed XDR phenotype, but no strain was resistant to all the tested antibiotics (**Figure 1**). The PCR assay results showed the presence of genes responsible for resistance to aminoglycosides, cephalosporins, carbapenems, and glycylcyclines. The ESBL β-lactamases, metallo β-lactamases, and other antibiotic resistance genes were detected, of which *bla*_CTX-M-15_ was the most common (32%; *n* = 40/126), followed by *bla*_SHV-11_ (27%; *n* = 34/126), *bla*_NDM-1_ (26%; *n* = 33/126), *bla*_NDM-5_ (16%; *n* = 20/126), *bla*_TEM-1_ (14%; *n* = 18/126), *bla*_CMY-2_ (13%; *n* = 16/126), *bla*_CTX-M-14_ (11%; *n* = 14/126), *bla*_VIM-2_ (9%; *n* = 12/126), *bla*_CTX-M-27_ and *bla*_GES-2_ (8%; *n* = 11/126 each), *bla*_CTX-M-9_ (6%; *n* = 8/126), and *bla*_KPC-2_ and *bla*_IMP-2_ (5%; *n* = 7/127 each). The aminoglycoside resistance gene *aac(3)IIIa* was found in 16% (*n* = 20/126) of the isolates, followed by *ant(2)Ib* in 8% (*n* = 10/126)*, aac(3)IIIb* in 5% (*n* = 7/126), and *aac(6)lia* in 3% (*n* = 4/126). Additionally, glycylcyclines resistance gene *tet* (A) was observed in 12% (*n* = 15/126) of the isolates (**Figure 2**).

### Sequence typing

**Figure 3:**
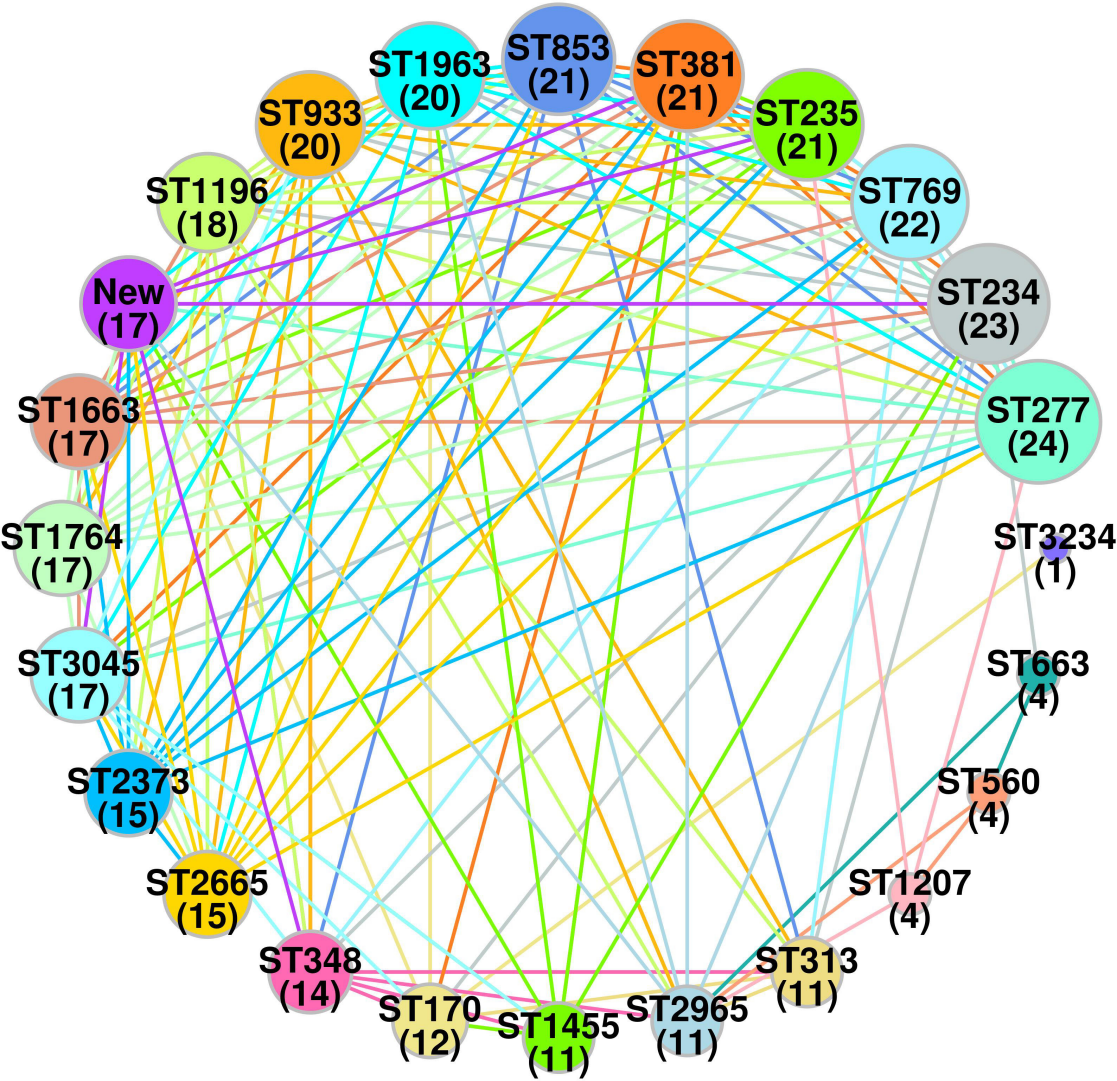
Spearman’s based co-relation network of sequence types and resistance phenotype among three groups (cephalosporin, carbapenem and aminoglycoside) in ESBL-producing *P. aeruginosa* (*n =* 126). Data were visualized by Gephi network analysis 0.94. The node size represents the STs of connection with other types. The edges represent the resistance patterns considered for the network. The thickness of the edge is proportional to the Spearman’s correlation coefficient (*ρ*) of the connection. The edge colors show significant relation, while the gray color does not show a significant correlation. Node: 1 (ST170); 2 (ST234); 3 (ST235); 4 (ST277); 5 (ST313); 6 (ST348); 7 (ST381); 8 (ST560); 9 (ST663); 10 (ST769); 11 (ST853); 12 (ST933); 13 (ST1196); 14 (ST1207); 15 (ST1455); 16 (ST1663); 17 (ST1764); 18 (ST1963); 19 (ST2373); 20 (ST2665); 21 (ST2965); 22 (ST3045); 23(new).

**Figure 4:**
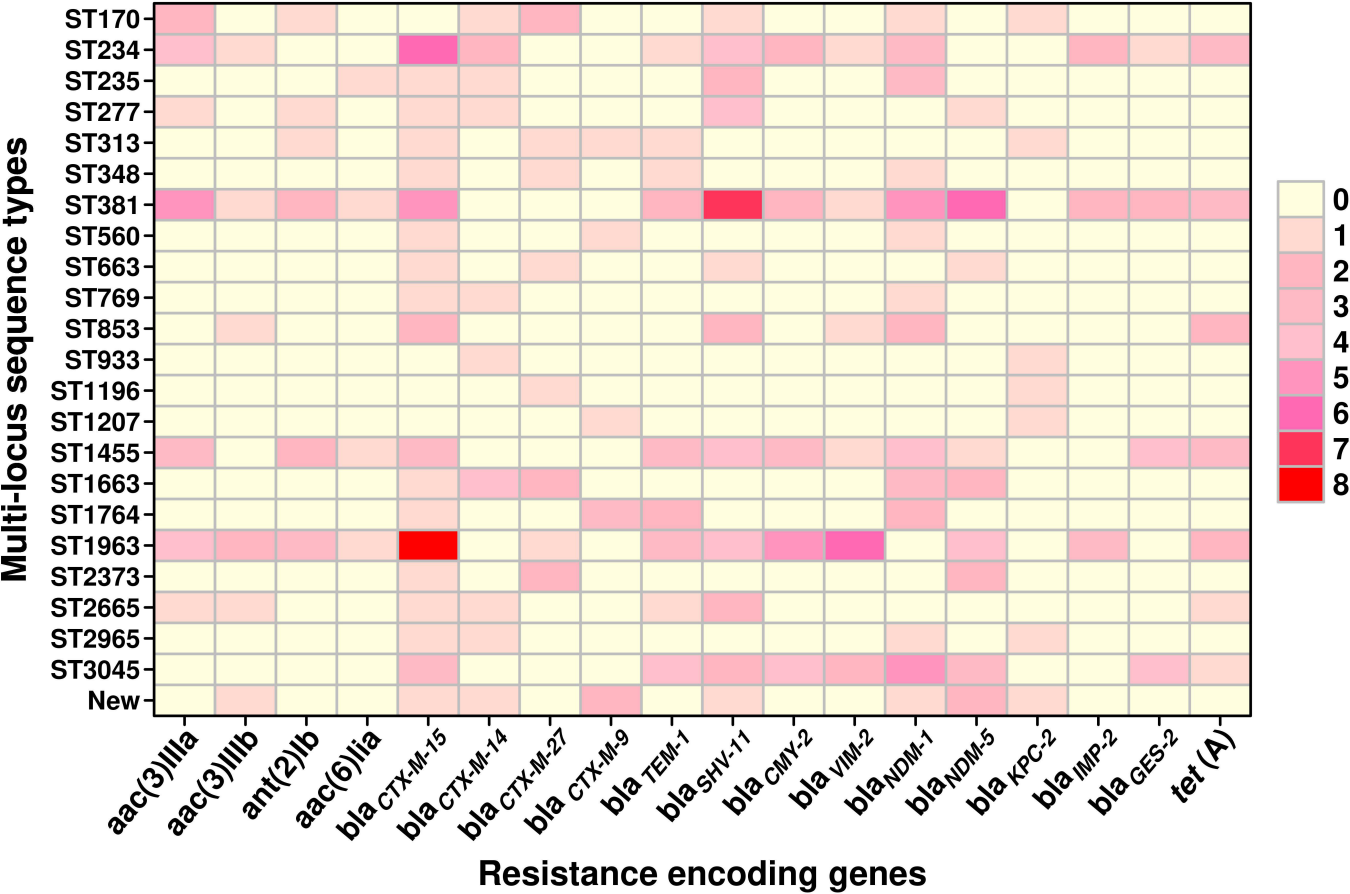
The graphical distribution of resistance-encoding genes associated with different sequence types in ESBL-producing *P. aeruginosa* (*n* = 126). The data were visualized using the PRISMA 8.0 software, which allows for the representation of the number of genes through the use of colors and shades. Lighter colors indicate a lower number of genes, while darker colors indicate a higher number of genes. The color gradient with increasing intensity indicates an increase in the number of genes.

A total of 23 STs were detected in the 126 screened isolates. These were ST1963 (12%; *n* = 16/126), followed by ST381 (11%; *n* = 14/126), ST234 (10%; *n* = 13/126), ST1455 (8%; *n* = 10/126), ST3045 (7%; *n* = 9/126), ST663 (5%; *n* = 7/126), and other STs, as shown in **Table 2**. A unique combination of alleles that did not yield a match in the published database was discovered, and this was designated as a “new” ST (**Supplementary Table 3**). We also analyzed the direct relation between STs and antimicrobial resistance groups. To answer this fundamental question, we performed Spearman’s correlation of all 23 STs and resistance antibiotic groups in 126 ESBL-producing *P. aeruginosa* isolates and visualized it by Gephi network analysis (**Figure 3**). The visualization network analyses revealed that ST234 and ST1663 had the greatest number of edges (*n* = 16), followed by ST933 and ST1196 (*n* = 13) (**Supplementary Table 4**). Further support analyses performed for all 23 STs and respective resistance antibiotic groups are presented in **Table 2**. The node and edge files were invariably attached, as demonstrated in **Supplementary Table 4**. The PRISMA 8.0 results uniquely show that *bla*_CTX-M-15_ was highly disseminated among the detected STs, followed by *bla*_SHV-11_ and *bla*_NDM-1_, but interestingly absent in the three isolates of strains ST933, ST1196, and ST1207. Meanwhile, *bla*_IMP,_ *bla*_GES,_ *tet* (A), and *aac(2,6)* were not prevalent in the ESBL-producing *P. aeruginosa.* The specific details about the dissemination of resistance genes in STs are shown in **Figure 4**.

**Table 2:** Frequency of sequence types (STs), antimicrobial susceptibility pattern, and plasmid characteristics.

Sr. no	STs	No. of strains, n (%)	Antimicrobial susceptibility to group specific				Incompatibility group (Inc) of plasmid	MOB subfamily (MOB-P, H, F)
			Aminoglycoside n (%)	Cephalosporin n (%)	Carbapenem n (%)	Glycylcycline n (%)		
1	ST170	3 (2.3)	3 (2.3)	3 (2.3)	3 (2.3)	0	IncColE, IncFI, IncHI, IncFIB	P51, F12, H11
2	ST234	13 (10.3)	4 (3.1)	12 (9.5)	13 (10.3)	3 (2.3)	IncFI, IncHI, IncL/M, IncA/C	F12, H11, P131, H121
3	ST235	4 (3.1)	1 (0.7)	3 (2.3)	3 (2.3)	0	IncHI, IncL/M	H11, P131
4	ST277	6 (4.7)	2 (1.5)	5 (3.9)	5 (3.9)	0	IncX, IncR, IncFIB	F12, P31, F11
5	ST313	5 (3.9)	1 (0.7)	4 (3.1)	4 (3.1)	0	IncX, IncI1	P31, P12
6	ST348	3 (2.3)	0	3 (2.3)	3 (2.3)	0	IncHI, IncFIS, IncL/M	F12, H11, P131
7	ST381	14 (11.1)	9 (7.1)	13 (10.3)	14 (11.1)	3 (2.3)	IncFI, IncX, IncA/C	F12, P31, H121
8	ST560	2 (1.5)	0	2 (1.5)	1 (0.7)	0	IncColE, IncI1	P51, P12
9	ST663	3 (2.35)	0	2 (1.5)	2 (1.5)	0	IncHI, IncFIS, IncL/M	H11, P131, F12
10	ST769	2 (1.5)	0	1 (0.7)	1 (0.7)	0	IncX, IncI1, IncFIB	P31, F12, P12
11	ST853	5 (3.9)	1 (0.7)	4 (3.1)	4 (3.1)	2 (1.5)	IncFI, IncFIS, IncL/M	F12, P131 P31, F12, F11
12	ST933	1 (0.7)	0	1 (0.7)	0	0	IncX, IncR, IncFIB
13	ST1196	1 (0.7)	0	1 (0.7)	1 (0.7)	0	IncColE, IncL/M, IncX,	P51, P131, F11 P31, P12, Q11
14	ST1207	1 (0.7)	0	1 (0.7)	1 (0.7)	0	IncX, IncI1, IncQ1
15	ST1455	10 (7.9)	6 (4.7)	10 (7.9)	10 (7.9)	3 (2.3)	IncFI, IncR, IncI1, IncFIB, IncA/C	H121, F12, F11, H121
16	ST1663	7 (4.7)	0	6 (4.7)	7 (4.7)	0	IncFI, IncHI, IncL/M, IncX	F12, H11, P31, P131
17	ST1764	6 (4.7)	0	6 (4.7)	6 (4.7)	0	IncFI, IncR, IncW	F12, F11
18	ST1963	16 (12.6)	10 (7.9)	16 (12.6)	16 (12.6)	2 (1.5)	IncFI, IncHI, IncFIS, IncX, IncFIB	F12, H11, P31, F11 H121, P131, P12
19	ST2373	4 (3.1)	0	3 (2.3)	4 (3.1)	0	IncL/M, IncI1, IncA/C
20	ST2665	5 (3.9)	2 (1.5)	5 (3.9)	5 (3.9)	1 (0.7)	IncHI, IncColE, IncX	P51, H11, P31
21	ST2965	2 (1.5)	0	1 (0.7)	1 (0.7)	0	IncFIS, IncColE	P51, F12
22	ST3045	9 (7.1)	0	9 (7.1)	9 (7.1)	1 (0.7)	IncFI, IncHI, IncL/M, IncX, IncFIB	F12, H11, P31, F12
23	New	4 (3.1)	1 (0.7)	4 (3.1)	4 (3.1)	0	IncFIS, IncX	P31, F12
	Total No.	126	40 (31.7)	115 (91.2)	117 (92.8)	15 (12%)		

### Plasmid analysis

The plasmid analysis was performed based on PCR replicon typing and MOB typing. The PCR-based replicon typing revealed that ESBL gene-positive *P. aeruginosa* consisted of plasmids belonging to the Incompatibility groups (Inc) IncColE, IncFI, IncHI, IncFIB, IncFIS, IncL/M, IncA/C, IncX, IncR, IncI1, and IncQ1 (**Table 2**). Furthermore, the plasmid MOB subgroup was determined. We found a major subfamily in common, which was MOBP: P12, P31, P51, and P131, followed by MOBH: H11 and H121; MOBF includes F11 and F12, while the MOBQ subfamily includes Q11. The PBRT and MOB typing results show that the ESBL-producing *P*. *aeruginosa* are key reservoirs for the diverse group of plasmids that are responsible for the dissemination of drug resistance determinants between *P*. *aeruginosa* and other species. The details of PBRT typing and its subfamily relation to the ST group are shown in **Table 2**. We successfully transferred ESBL-encoding genes carried on plasmids to *E. coli* C_600_ with a success rate of 66.66% (80/126). The frequency of transfer ranged from 10^−5^ to 10^−3^.

## Discussion

*P. aeruginosa* is a significant opportunistic pathogen and a leading cause of hospital-acquired infections worldwide. Its high level of antibiotic resistance has made it a major public health concern (Glen and Lamont, 2021). Antimicrobial agents, including aminoglycosides, carbapenems, cephalosporins, fluoroquinolones, monobactams, and polymyxins, are used to treat *P. aeruginosa* infections. However, this pathogen can rapidly develop resistance to antimicrobials through chromosomal mutations or the horizontal acquisition of resistant determinants (Horcajada et al., 2019). *P. aeruginosa* infections have periodically increased in China, and the pathogen has shown a persistent resistance to commonly used antimicrobials (Ding et al., 2016). The main objective of this study was to determine the prevalence and antimicrobial susceptibility of ESBLs and other antibiotic resistance determinants in *P. aeruginosa* isolated from pediatric patients. We analyzed *P. aeruginosa* infections based on sex, specimen, and department, but no significant difference was found. However, the 1–6 years age group was identified as a significant age group for *P. aeruginosa* infection, which is consistent with the existing literature (Douglas et al., 2009). It is important to note that the development of ESBL-producing *P. aeruginosa* is multifactorial and can occur due to factors such as prolonged hospital stay, frequent antibiotic use, and exposure to invasive medical procedures. In this study, the higher rate of ESBL production in hospitalized departments could be due to the frequent use of antibiotics to avoid co-infection, which can lead to the selection of resistant strains (Fernández-Barat et al., 2017). In contrast, outpatient department patients may not explore antimicrobials as they are young children, which could have reduced the likelihood of ESBL production. However, this study did not investigate the specific factors contributing to the development of ESBL-producing *P. aeruginosa* in these patient groups, and further research is needed to fully understand the underlying mechanisms, such as clinical conditions, hospital stay, and antimicrobial therapy over 30 days, which are all important factors that should be considered in future studies. The identification of *P. aeruginosa* using both the VITEK^®^2 compact system and API-20 did not yield significantly different results (*p-*value = 0.56), suggesting that both methods are reliable. A previous comparative study reported that the VITEK^®^2 compact system demonstrated 92% accuracy in identifying *P. aeruginosa*, which is consistent with the results obtained in our study (Joyanes et al., 2001). We observed high levels of resistance among commonly used antimicrobials, including piperacillin–tazobactam (56%), cefepime (40%), ceftazidime (39%), imipenem (36%), and ciprofloxacin (32%). These findings are consistent with the resistance patterns of *P. aeruginosa* strains reported globally and in other provinces of China, such as Zhejiang, and the rates of resistance are increasing rapidly. This trend is particularly concerning for the healthcare sector (Glupczynski et al., 2010; Lin et al., 2022). Furthermore, the Vitek^®^2 compact system (BioMerieux, France) and DDDM identified 42% of *P. aeruginosa* isolates as ESBL producers, which is lower than the reported rates in China but comparable to other Asian countries like Malaysia (Chen et al., 2015; Dwiyanto et al., 2022). We reported that 10% of our isolates co-produced ESBL and MBL, whereas in the neighboring country of Nepal, the reported percentage was 3.1%, indicating a lower prevalence compared to our finding (Shrestha et al., 2022), due to the changes in epidemiology related to the clones circulating in different countries. The higher prevalence of ESBL and MBL co-producers in China indicates that more measures need to be in place for the early detection of strains and to limit their clonal spread. By doing so, it can greatly decrease the burden of infectious disease and have positive outcomes on patient mortality and morbidity. We have demonstrated that both methods are potentially effective for detecting the ESBL enzyme in *P. aeruginosa*. PCR assay and sequencing have confirmed the presence of resistance determinants in ESBL-producing *P. aeruginosa* including *bla*_CTX-M-15_ (32%) and *bla*_SHV-11_ (27%), carbapenem resistance *bla*_NDM-1_ (26%), *bla*_NDM-M-5_ (16%), aminoglycoside resistance *aac(3)IIIa* (16%), and glycylcyclines resistance *tet* (A) (12%). We report that *bla*_CTX-M-15_ is the predominant ESBL-encoding gene present in *P. aeruginosa* and is highly disseminated in the reported “23” STs. Similar resistance-encoding genes were reported globally, including in China, but our study is in contrast with some reports in China (Chen et al., 2015; Hosu et al., 2021; Dwiyanto et al., 2022; Lin et al., 2022). No *bla*_OXA-48_ gene was reported in this study. Our concern is the occurrence of the co-existence of the ESBLs and carbapenemase genes in *P. aeruginosa*, which can render the bacterium MDR or XDR. The present study does not prove the co-existence of the location of such genes on a single plasmid, but they can transfer easily to other strains with plasmid transmission frequencies ranging from 10^−5^ to 10^−3^. It is noteworthy that among all the ESBL-producing *P. aeruginosa* isolates, we identified 23 sequence types and one previously unknown type among the 126 samples. Our results from multi-locus sequence typing (MLST) revealed that the ST1963 strain was the most prevalent circulating type, followed by ST381, ST234, ST235, ST1455, ST3045, and ST663. All of the ST types of *P. aeruginosa* that we identified in our study have been previously reported in China, with ST235 and ST1963 being the dominant types (Fan et al., 2016). ST235 is recognized as a globally distributed drug-resistant lineage, as it has been predominantly associated with nosocomial outbreaks (Maatallah et al., 2011). It is worth noting that our ST235 isolates were also resistant to fluoroquinolones (FQ), and they likely evolved as a result of FQ usage. FQs have been used as an antipseudomonal treatment since 1984 in Europe (Hsu et al., 2005). ST1963 was initially identified in Kunming, China, along with the presence of *bla*_KPC-2_ carbapenemase. This region is geographically close to Shenzhen, suggesting the potential spread of this clone in the surrounding regions. In our study, all the ST381 and ST234 isolates were found to be resistant to carbapenems, which is consistent with the findings of a prior study (Chen et al., 2022). In another study, it was found that the *P. aeruginosa* strain BH9 belonging to ST381 was positive for the *bla*_KPC-2_ carbapenemase that was present on a plasmid that resembles a phage. However, in our study, the ST381 isolates we identified possessed either *bla*_NDM-1_ (*n* = 6) or *bla*_NDM-5_ (*n* = 7) carbapenemase instead (Wang et al., 2021). An earlier report from a tertiary hospital in Daejeon, Korea, indicated the absence of any resistance genes in ST1455. However, in our study, all of the ST1455 isolates were found to be resistant to carbapenems (Cho et al., 2015). Carbapenem resistance in ST3045 isolates has been previously reported in dogs in Japan. However, in our study, carbapenem-resistant ST3045 isolates were found in human hosts (Hayashi et al., 2021). *P. aeruginosa* strain ST663 has been observed in France and has also been isolated from healthy human volunteers in Spain, where it has an imipenem breakpoint of 4 mg/L, which groups it into the sensitive categories (Balabanova et al., 2020). The majority of our ST663 isolates (67%) demonstrated resistance to carbapenems. Of the 12% (*n* = 16/126) isolates that belonged to ST1963, half of them carried *bla*_CTX-M-15_. These findings suggest that *P. aeruginosa* ST1963 harboring ESBL genes may be widely circulating in southern China and other geographical areas; thus, this requires further investigation. Notably, ST292 has been reported in southern China and neighboring countries such as Thailand, but we did not observe it in our study (Maatallah et al., 2011; Khuntayaporn et al., 2019). Furthermore, our network analyses showed that ST663, ST1763, and ST1963 were interconnected and had similar resistance profiles, suggesting the potential spread of antibiotic-resistance genes between these strains. The transfer of resistance genes between different strains is often facilitated by transmissible plasmids that belong to different Inc categories based on replicon typing, which prevents the co-existence of related plasmids in the same cell. In this study, we observed that ESBL-positive *P. aeruginosa* carries a diverse range of plasmids belonging to Inc categories such as IncColE, IncFI, IncHI, IncFIB, IncFIS, IncL/M, IncA/C, IncX, IncR, IncI1, and IncQ1. Additionally, our analysis of the MOB subfamily data revealed that the plasmids belonged to four subfamilies: MOBP (P12, P31, P51, and P131), MOBH (H11 and H121), MOBF (F11 and F12), and MOBQ11. Previous studies have also reported the high prevalence of IncF and IncH replicon types of plasmids in ESBL-positive *P. aeruginosa*, which is consistent with the findings of our study (Cejas et al., 2022). We also observed highly transferable replicon types such as IncF, IncH, and IncX along with the MOBP subfamily in our isolates. There is very little information on the prevalence of *P. aeruginosa* in pediatric patients, and most of the published research is on adult patients. To gain a comprehensive view of the evolution of strains in this study, further analyses can be performed using whole-genome sequencing analyses. Conjugation studies can also aid in understanding the spread of resistance genes in clones of *P. aeruginosa*. Our study confirms that the use of β-lactam antibiotics, particularly cephalosporins and carbapenems, has a direct impact on the development of resistance in hospital settings. Therefore, it is essential to monitor and use antibiotics prudently and track the evolution of novel variants of *P. aeruginosa*, which can develop into MDR clones. It is important to screen for specific antibiotic resistance-encoding genes such as *bla*_CTX-M_, *bla*_SHV_, and *bla*_NDM_, as they play an important role in the development of MDR in *P. aeruginosa*. Further surveillance should provide reliable baseline data for monitoring and controlling any emergence of multidrug-resistant clones of *P. aeruginosa* in Shenzhen. It will also help in developing policies on the clinical usage of antibiotics in pediatric settings. The present study has several limitations that need to be acknowledged. Firstly, the phenotypic detection of carbapenemase production was not included in the study, which restricts our ability to fully characterize the carbapenem resistance mechanisms in the ESBL-producing isolates. Additionally, the study focused solely on ESBL detection and did not include other significant resistance mechanisms, such as *AmpC* β-lactamase production or efflux pump activity. The present study can be further extended by screening for mutations and genes conferring colistin resistance in these isolates, which is one of the last-resort drugs used in the treatment of Gram-negative infections. Our study provides an indication on the south China region and serves as a reference for any upcoming national or global studies that may indeed provide a wider perspective on the epidemiology of disease.

## Conclusions

Based on the results of this study, it can be concluded that multidrug-resistant *P. aeruginosa* poses a significant threat to young children in clinical settings in China. The high level of resistance rates observed for commonly used antibiotics, such as piperacillin–tazobactam, cefepime, ceftazidime, and carbapenems, highlights the urgent need to develop novel or alternative therapies for the treatment of disease. The prevalence of ESBL-producing *P. aeruginosa*, particularly the presence of the *bla*_CTX-M-15_ and *bla*_NDM-1_ genes, underscores the importance of active surveillance and infection control measures to prevent the spread of these resistant strains. Antibiotic resistance in *P. aeruginosa* is likely spread by both clonal dissemination and horizontal transfer of resistance genes *via* insertion sequences, plasmids, and other mobile genetic elements. The existence of different plasmid types and incompatibility groups shows the complexity of resistance mechanisms and the need for more research to help us comprehend the dynamics of the spread of antimicrobial resistance determinants. Overall, this study highlights the importance of continued surveillance and monitoring of antibiotic resistance in *P. aeruginosa*, particularly in vulnerable populations such as young children. Effective infection control measures and the development of new treatment options are urgently needed to combat the emergence and spread of multidrug-resistant *P. aeruginosa*.

## Data availability statement

The raw data supporting the conclusions of this article will be made available by the authors, without undue reservation.

## Ethics statement

This study was conducted according to the guidelines of the Declaration of Helsinki and approved by the Institutional Ethics Committee, Shenzhen Children’s Hospital, reference number: 2018 (013), dated 3rd Sept 2018, which complies with international ethical standards.

## Author contributions

All authors contributed to the data analysis, drafting and revising the article, have agreed to the final approval of the version to be published, and agree to be held accountable for all aspects of the work.
