# Supplementary material for: Resistance genomics and molecular epidemiology of high-risk clones of ESBL-producing Pseudomonas aeruginosa in young children
Source: Front Cell Infect Microbiol. 2023 May 24;13:1168096. doi: 10.3389/fcimb.2023.1168096 (PMC10244630; doi:10.3389/fcimb.2023.1168096)
Supplement: Supplementary file 1 [file DataSheet_1.pdf]

Supplementary Material

Resistance genomics and molecular epidemiology of High-risk clones of ESBLs producing *Pseudomonas aeruginosa* in young children

Sandip Patil[1,2], Xiaowen Chen[2], Shaowei Dong[2], Huirong Mai[1], Bruno Silvester Lopes[3,4], Sixi Liu[1*] and Feiqiu Wen[1,2*]

*** Correspondence:**

Prof. Feiqiu Wen, MD, PhD

Department of Haematology and Oncology,

Shenzhen Children's Hospital, Shenzhen, Guangdong, 518038, China.

Ph: +86-755-83009888, E-mail: fwen62@163.com

Supplementary Table 1: Primers in this study for β-lactamase encoding gene detection, melting temperature and amplicon size;

Supplementary Table 2: Primers in this study for *P. aeruginosa* MLST scheme, melting temperature and amplicon size;

Supplementary Table 3: Novel STs allelic combination for new strain;

Supplementary Table 4: The node and edge used for determining the relation between the antibiotic groups and STs

Supplementary Table 1: Primers in this study for β-lactamase encoding gene detection, melting temperature and amplicon size

List of primers used for the detection of β-lactamase genes				
Sr No	Gene	Primer	Products Size (bp)	Tm (°C)
1	*bla*$_{CTX-M}$	F-5′-TTTGCGATGTGCAGTACCAGTAA-3′<br>R-5′-CGATATCGTTGGTGGTGCCATA-3′	554	55
2	*bla*$_{VIM}$	F- 5'-ATTCCGGTCGG(A/G)GAGGTCCG-3'<br>R-5'-GAGCAAGTCTAGACCGCCCG-3'	633	54
3	*bla*$_{KPC}$	F-5'-ATGTCACTGTATCGCCGTC-3'<br>R-5'-TTACTGCCCGTTAACGCC-3'	883	56
4	*bla*$_{NDM}$	F-5'-TGCGGGGTTTTTAATGCTG-3'<br>R-5'-TGGCTCATCACGATCATGC-3'	785	56
5	*bla*$_{IMP}$	F-5'-GTTAAAAGTTATTAGTAGTTTATTG-3'<br>R-5'-CTACTCGGCGACTGAGC-3'	799	55
6	*bla*$_{OXA}$	F-5'-TTGGTGGCATCGATTATCGG-3'<br>R-5'-GAGCACTTCTTTTGTGATGGC-3'	745	56
7	*bla*$_{SHV}$	F-5'-GGCTATGCGTTATATTCGCC-3’<br>R-5'-GGTTAGCGTTGCCAGTGC-3’	865	56
8	*bla*$_{GES}$	F-5'-ATGCGCTTCATTCACGCAC-3’<br>R-5'-CTATTTGTCCGTGCTCAGG-3’	846	57
9	*bla*$_{TEM}$	F-5'-AGGAAGAGTATGATTCAACA-3’<br>R-5'-CTCGTCGTTTGGTATGGC-3’	535	56
10	*bla*$_{CMY}$	F-5'- CTGACAGCCTCTTTCTCCA-3'<br>F-5'- GCCAAACAGACCAATGCT-3'	504	55

Supplementary Table 2: Primers in this study for *P. aeruginosa* MLST scheme, melting temperature and amplicon size

The primers used for the amplification of housekeeping genes to determine MLST profile					
**No**	**Target**	**Primer**	**Primer sequences**	**Tm (°C)**	**Product Size (bp)**
1	Acetyl coenzyme A synthetase	*acsA*	F-5'-ACCTGGTGTACGCCTCGCTGAC-3'<br>R-5'-GACATAGATGCCCTGCCCCTTGAT-3'	55	842
2	Shikimate dehydrogenase	*aroE*	F-5'-TGGGGCTATGACTGGAAACC-3'<br>R-5'TAACCCGGTTTTGTGATTCCTACA-3'	55	825
3	GMP synthase	*guaA*	F-5'-CGGCCTCGACGTGTGGATGA-3'<br>R-5'-GAACGCCTGGCTGGTCTTGTGGTA-3'	55	940
4	DNA mismatch repair protein	*mutL*	F-5'-CCAGATCGCCGCCGGTGAGGTG-3'<br>R-5'-CAGGGTGCCATAGAGGAAGTC-3'	55	940
5	NADH dehydrogenase I chain C, D	*nuoD*	F-5'-ACCGCCACCCGTACTG-3'<br>R-5'-TCTCGCCCATCTTGACCA-3'	55	1042
6	Phosphoenolpyruvate synthase	*ppsA*	F-5'-GGTCGCTCGGTCAAGGTAGTGG-3'<br>R-5'-GGGTTCTCTTCTTCCGGCTCGTAG-3'	55	989
7	Anthralite synthetase component I	*tepE*	F-5'-GCGGCCCAGGGTCGTGAG-3'<br>R-5' CCCGGCGCTTGTTGATGGTT-3'	55	811

Continue ……

The primers used for the PCR based replicon typing				
**Name**	**DNA sequence (5'-3')**	**Target site**	**EMBL accession no.**	**Product size (bp)**
Hll FW	GGAGCGATGGATTACTTCAGTAC	parA -parB	AF250878	471
Hll RV	TGCCGTTTCACCTCGTGAGTA			
Hl2 FW	TTTCTCCTGAGTCACCTGTTAACAC	iterons	BX664015	644
Hl2 RV	GGCTCACTACCGTTGTCATCCT			
II FW	CGAAAGCCGGACGGCAGAA	RNAJ	M20413	139
II RV	TCGTCGTTCCGCCAAGTTCGT			
X FW	AACCTTAGAGGCTATTTAAGTTGCTGAT	ori 'I	Y00768	376
X RV	TGAGAGTCAATTTTTATCTCATGMTAGC			
UM FW	GGATGAAAACTATCAGCATCTGAAG	repA,B,C	U27345	785
UM RV	CTGCAGGGGCGATTCMAGG			
N FW	GTCTAACGAGCTTACCGAAG	repA	NC_003292	559
N RV	GTTTCAACTCTGCCAAGTTC			
FIA FW	CCATGCTGGTTCTAGAGAAGGTG	iterons	101724	462
FIA RV	GTATATCCTTACTGGCTTCCGCAG			
FIB FW	GGAGTTCTGACACACGATTTTCTG	repA	M26308	702
FIB RV	CTCCCGTCGCTTCAGGGCATT			
W FW	CCTAAGAACAACAAAGCCCCCG	repA	U l 2441	242
W RV	GGTGCGCGGCATAGAACCGT			
Y FW	AATTCAAACAACACTGTGCAGCCTG	repA	K02380	765
Y RV	GCGAGAATGGACGATTACAAAACTTT			
P FW	CTATGGCCCTGCAAACGCGCCAGAAA	iterons	M20134	534
P RV	TCACGCGCCAGGGCGCAGCC			
FIC FW	GTGAACTGGCAGATGAGGAAG G	repA2	AH003523	262
FIC RV	TTCTCCTCGTCGCCAAACTAGAT			
A/C FW	GAGAACCAAAGACAAAGACCTGGA	repA	X73674	465
A/C RV	ACGACAAACCTGAATTGCCTCCTT			
T FW	TTGGCCTGTTTGTGCCTAAACCAT	repA	K00053	750
T RV	CGTTGATTACACTTAGCTTTGGAC			
FIIs FW	CTGTCGTAAGCTGATGGC	repA	AE006471	270
FIIs RV	CTCTGCCACAAACTTCAGC			

FrcpB FW	TGATCGTTTAAGGAAMTG	RNAl/repA	AY234375	270
FrcpB RV	GCGGTCCGGAAAGCCAGAAAAC			160
K/B FW	GAAGATCAGTCACACCATCC	RNAI	M93063	
KRV	TCTTTCACGAGCCCGCCAAA			
B/O RV	TCTGCGTTCCGCCAAGTTCGA	RNAI	M28718	159

Continue ……..

frontiers

List of degenerate primers used for Degenerate Primer MOB Typing (DPMT)				
**Primer name**	**Primer sequence (5'-3')**	**PCR conditions**	**Prototype**	**Amplicon size (bp)**
F11-f	GCA GCG TAT TAC TTC TCT GCT GCC GAY GAY TAY TA	25 cycles, 53˚C	R388	234
F1-r	ACT TTT GGG CGC GGA RAA BTG SAG RTC			
F12-f	AGC GAC GGC AAT TAT TAC ACC GAC AAG GAY AAY TAY TA	25 cycles, 55°C	F	234
F1-r	ACT TTT GGG CGC GGA RAA BTG SAG RTC			
P11-f	CGT GCG AAG GGC GAC AAR ACB TAY CA	25 cycles, 60°C	RP4	180
P1-r	AGC GAT GTG GAT GTG AAG GTT RTC NGT RTC			
P12-f	GCA CAC TAT GCA AAA GAT GAT ACT GAY CCY GTT TT	30 cycles, 53.8°CC, 1.5U Taq per reaction	R64	189
P1-r	AGC GAT GTG GAT GTG AAG GTT RTC NGT RTC			
P131-f	AAC CCA CGC TGC AAR GAY CCV GT	30 cycles, 59°C, 15 seconds of extension per cycle	pCTX-M3	180
P1-r	AGC GAT GTG GAT GTG AAG GTT RTC NGT RTC			
P14-f	CGC AGC AAG GAC ACC ATC AAY CAY TAY RT	25 cycles, 50°C	pRAS3.1	174
P1-r	AGC GAT GTG GAT GTG AAG GTT RTC NGT RTC			
P3-f	CC GTG AGC CAA ATC ACA CAG AAT ATK RTB TT	25 cycles, 50°C	R6K	177
P3-r	CG AAA GCC AAC ATG AAC ATG HGG ATK HTC			
P4-f	GCG TTC AGG ATG GTC YTB TCS ATG CC	25 cycles, 64°C	pRA3	163
P4-r	C GGT TTT GAC CGT CAG ATG SVM ATG CGG			
P51-f	T ACC ACG CCC TAT GCG AAR AAR TAY AC	30 cycles, 58°C, 20 seconds of extension per cycle	ColE1	167
P5-r	CC CTT GTC CTG GTG YTS NAC CCA			

P52-f	GAT AGC CTT GAT TTT AAT AAC ACC AAY ACY TAY AC	30 cycles, 58°C, 20 seconds of extension per cycle	p9555	175
P5-r	CC CTT GTC CTG GTG YTS NAC CCA			
P53-f	G GGC TCG CAC GAY CAY ACN GG	30 cycles, 65°C	pAsal1	345
P53-r	GC CCA GCC CTT TTC RTG RTT RTG			
Q11-f	CAA TCG TCC AAG GCG AAR GCN GAY TA	30 cycles, 50°C	RSF1010	331
Q11-r	CG CTC GGA GAT CAT CAY YTG YCA YTG			
Q12-f	CTG GAA TAT ACT GAA CAC GGN AAY ATG CC	30 cycles, 52°C	pP	341
Q12-r	ATC CTT GGT GTT AGC ACG TTT RAA RWA YTG			
Qu-f	AGC GCC GTG CTG TCC GCB GCN TAY CG	30 cycles, 64°C	pIGWZ12	179
Qu-r	CTC CGC AGC CTC GRC SGC RTT CCA			
H11-f	CCG GCG TCG GAG AAY CAY CAY CA	Touchdown PCR: start at 65°C DTa = 21uC per cycle, 15 cycles at 55uC	R27	207
H11-r	AAG GTC GTA TAC CTT YCC KGC RTC RTG			
H121-f	G CCA GCT TCC GAA TCA CAY CAY CAY CG	25 cycles, 59°C	pSN254	313
H121-r	G TCG CTT GTC GCG CCA CCG DAT RAA RTA			
H2-f	AG TTC CCA GCC TCA GAA ATC CAY CAY CAY KC	25 cycles, 68°C	pKLC102	264
H2-r	G CGG ACC GTG CCA NGG RTG CCA			
C11-f	GT CAG GTC AGC GTG TGG GGN CTN AC	Touchdown PCR: start at 65°C DTa = 21uC per cycle, 20 cycles at 55uC	CloDF13	283
C11-r	CT CTT CAC GGT GCC CTC NAC YTC RAA			
C12-f	GC ACG ACT GGA AAA ATA TCG CTA TGG GGN ATH AC	30 cycles, 59°C	p29930	257
C12-r	CAA CGT GAT AAT CCC GTC RGG VCG RTG			

Supplementary Table 3: Novel STs allelic combination for a new strain

new allelic combination for novel ST type in present study								
Locus	*acsA*	*aroE*	*guaA*	*mutL*	*nuoD*	*ppsA*	*trpE*	ST
Allelic combination	15	4	11	5	4	3	141	New

Supplementary Table 4: The node and edge used for determining the relation between the antibiotic groups and STs

The node and edge used for determining the relation between the antibiotic groups and STs									
Id	Label	Node					ID	Label	number of edges
1	ST170	Source	Target	Type	From	TO	2	ST234	16
2	ST234	1	2	unidirectional	ST170	ST3234	16	ST1663	16
3	ST235	1	7	unidirectional	ST170	ST381	12	ST933	13
4	ST277	1	15	unidirectional	ST170	ST1455	13	ST1196	13
5	ST313	1	18	unidirectional	ST170	ST1963	4	ST277	11
6	ST348	1	22	unidirectional	ST170	ST3045	3	ST235	10
7	ST381	1	23	unidirectional	ST170	New	10	ST769	10
8	ST560	2	1	unidirectional	ST234	ST170	11	ST853	10
9	ST663	2	4	unidirectional	ST234	ST277	17	ST1764	10
10	ST769	2	5	unidirectional	ST234	ST313	7	ST381	9
11	ST853	2	6	unidirectional	ST234	ST348	18	ST1963	9
12	ST933	2	7	unidirectional	ST234	ST381	19	ST2373	9
13	ST1196	2	9	unidirectional	ST234	ST663	20	ST2665	9
14	ST1207	2	10	unidirectional	ST234	ST769	1	ST170	6
15	ST1455	2	11	unidirectional	ST234	ST853	6	ST348	6
16	ST1663	2	12	unidirectional	ST234	ST933	15	ST1455	6
17	ST1764	2	13	unidirectional	ST234	ST1196	5	ST313	5
18	ST1963	2	15	unidirectional	ST234	ST1455	22	ST3045	5
19	ST2373	2	16	unidirectional	ST234	ST1663	23	New	5
20	ST2665	2	17	unidirectional	ST234	ST1764	21	ST2965	4
21	ST2965	2	18	unidirectional	ST234	ST1963	8	ST560	3
22	ST3045	2	22	unidirectional	ST234	ST3045	14	ST1207	3

23	New	2	23	unidirectional	ST234	New	9	ST663	2
		3	4	unidirectional	ST235	ST277			
		3	7	unidirectional	ST235	ST381			
		3	10	unidirectional	ST235	ST769			
		3	11	unidirectional	ST235	ST853			
		3	16	unidirectional	ST235	ST1663			
		3	17	unidirectional	ST235	ST1764			
		3	18	unidirectional	ST235	ST1963			
		3	19	unidirectional	ST235	ST2373			
		3	22	unidirectional	ST235	ST3045			
		3	23	unidirectional	ST235	New			
		4	2	unidirectional	ST277	ST234			
		4	3	unidirectional	ST277	ST235			
		4	7	unidirectional	ST277	ST381			
		4	10	unidirectional	ST277	ST769			
		4	11	unidirectional	ST277	ST853			
		4	16	unidirectional	ST277	ST1663			
		4	17	unidirectional	ST277	ST1764			
		4	18	unidirectional	ST277	ST1963			
		4	19	unidirectional	ST277	ST2373			
		4	22	unidirectional	ST277	ST3045			
		4	23	unidirectional	ST277	New			
		5	1	unidirectional	ST313	ST170			
		5	6	unidirectional	ST313	ST348			
		5	12	unidirectional	ST313	ST933			
		5	13	unidirectional	ST313	ST1196			

5	21	unidirectional	ST313	ST2965
6	1	unidirectional	ST348	ST170
6	5	unidirectional	ST348	ST313
6	12	unidirectional	ST348	ST933
6	13	unidirectional	ST348	ST1196
6	15	unidirectional	ST348	ST1455
6	21	unidirectional	ST348	ST2965
7	1	unidirectional	ST381	ST170
7	2	unidirectional	ST381	ST234
7	4	unidirectional	ST381	ST277
7	10	unidirectional	ST381	ST769
7	11	unidirectional	ST381	ST853
7	15	unidirectional	ST381	ST1455
7	18	unidirectional	ST381	ST1963
7	22	unidirectional	ST381	ST3045
7	23	unidirectional	ST381	New
8	9	unidirectional	ST560	ST663
8	14	unidirectional	ST560	ST1207
8	21	unidirectional	ST560	ST2965
9	8	unidirectional	ST663	ST560
9	21	unidirectional	ST663	ST2965
10	3	unidirectional	ST769	ST235
10	4	unidirectional	ST769	ST277
10	5	unidirectional	ST769	ST313
10	6	unidirectional	ST769	ST348
10	11	unidirectional	ST769	ST853

10	12	unidirectional	ST769	ST933
10	16	unidirectional	ST769	ST1663
10	17	unidirectional	ST769	ST1764
10	18	unidirectional	ST769	ST1963
10	19	unidirectional	ST769	ST2373
11	3	unidirectional	ST853	ST235
11	4	unidirectional	ST853	ST277
11	5	unidirectional	ST853	ST313
11	6	unidirectional	ST853	ST348
11	10	unidirectional	ST853	ST769
11	12	unidirectional	ST853	ST933
11	16	unidirectional	ST853	ST1663
11	17	unidirectional	ST853	ST1764
11	18	unidirectional	ST853	ST1963
11	19	unidirectional	ST853	ST2373
12	3	unidirectional	ST933	ST235
12	4	unidirectional	ST933	ST277
12	5	unidirectional	ST933	ST313
12	6	unidirectional	ST933	ST348
12	10	unidirectional	ST933	ST769
12	11	unidirectional	ST933	ST853
12	13	unidirectional	ST933	ST1196
12	16	unidirectional	ST933	ST1663
12	17	unidirectional	ST933	ST1764
12	18	unidirectional	ST933	ST1963
12	19	unidirectional	ST933	ST2373

12	20	unidirectional	ST933	ST2665
12	21	unidirectional	ST933	ST2965
13	3	unidirectional	ST1196	ST235
13	4	unidirectional	ST1196	ST277
13	5	unidirectional	ST1196	ST313
13	6	unidirectional	ST1196	ST348
13	10	unidirectional	ST1196	ST769
13	11	unidirectional	ST1196	ST853
13	12	unidirectional	ST1196	ST933
13	16	unidirectional	ST1196	ST1663
13	17	unidirectional	ST1196	ST1764
13	18	unidirectional	ST1196	ST1963
13	19	unidirectional	ST1196	ST2373
13	20	unidirectional	ST1196	ST2665
13	21	unidirectional	ST1196	ST2965
14	3	unidirectional	ST1207	ST235
14	4	unidirectional	ST1207	ST277
14	21	unidirectional	ST1207	ST2965
15	1	unidirectional	ST1455	ST170
15	2	unidirectional	ST1455	ST234
15	7	unidirectional	ST1455	ST381
15	18	unidirectional	ST1455	ST1963
15	22	unidirectional	ST1455	ST3045
15	23	unidirectional	ST1455	New
16	2	unidirectional	ST1663	ST234
16	4	unidirectional	ST1663	ST277

16	7	unidirectional	ST1663	ST381
16	10	unidirectional	ST1663	ST769
16	13	unidirectional	ST1663	ST1196
16	21	unidirectional	ST1663	ST3045
17	2	unidirectional	ST1764	ST234
17	3	unidirectional	ST1764	ST235
17	4	unidirectional	ST1764	ST277
17	7	unidirectional	ST1764	ST381
17	10	unidirectional	ST1764	ST769
17	11	unidirectional	ST1764	ST853
17	16	unidirectional	ST1764	ST1663
17	20	unidirectional	ST1764	ST2665
17	22	unidirectional	ST1764	ST3045
17	23	unidirectional	ST1764	New
18	3	unidirectional	ST1963	ST235
18	4	unidirectional	ST1963	ST277
18	7	unidirectional	ST1963	ST381
18	10	unidirectional	ST1963	ST769
18	11	unidirectional	ST1963	ST853
18	16	unidirectional	ST1963	ST1663
18	20	unidirectional	ST1963	ST2665
18	22	unidirectional	ST1963	ST3045
18	23	unidirectional	ST1963	New
19	3	unidirectional	ST2373	ST235
19	4	unidirectional	ST2373	ST277
19	7	unidirectional	ST2373	ST381

19	10	unidirectional	ST2373	ST769
19	11	unidirectional	ST2373	ST853
19	16	unidirectional	ST2373	ST1663
19	20	unidirectional	ST2373	ST2665
19	22	unidirectional	ST2373	ST3045
19	23	unidirectional	ST2373	New
20	3	unidirectional	ST2665	ST235
20	4	unidirectional	ST2665	ST277
20	7	unidirectional	ST2665	ST381
20	10	unidirectional	ST2665	ST769
20	11	unidirectional	ST2665	ST853
20	16	unidirectional	ST2665	ST1663
20	20	unidirectional	ST2665	ST2665
20	22	unidirectional	ST2665	ST3045
20	23	unidirectional	ST2665	New
21	2	unidirectional	ST2965	ST234
21	7	unidirectional	ST2965	ST381
21	18	unidirectional	ST2965	ST1963
21	23	unidirectional	ST2965	New
22	1	unidirectional	ST3045	ST170
22	6	unidirectional	ST3045	ST348
22	12	unidirectional	ST3045	ST933
22	15	unidirectional	ST3045	ST1455
22	23	unidirectional	ST3045	New
23	2	unidirectional	New	ST234
23	3	unidirectional	New	ST235

23	6	unidirectional	New	ST348
23	7	unidirectional	New	ST381
23	22	unidirectional	New	ST3045
